# Cross-Culture Adaptation and Psychometric Properties of the DrInC Questionnaire in Tanzanian Swahili

**DOI:** 10.3389/fpubh.2018.00330

**Published:** 2018-11-20

**Authors:** Duan Zhao, Catherine A. Staton, Qing He, Blandina Theophil Mmbaga, Joao Ricardo Nickenig Vissoci

**Affiliations:** ^1^Duke Kunshan Global Health Research Center, Duke Kunshan University, Kunshan, China; ^2^Duke Global Health Institute, Duke University, Durham, NC, United States; ^3^Department of Linguistics, South China University of Technology, Guangzhou, China; ^4^Kilimanjaro Christian Medical Centre, Moshi, Tanzania; ^5^Research and Innovation, Kilimanjaro Clinical Research Institute, Moshi, Tanzania; ^6^Research and Innovation and Paediatric and Child Health, Kilimanjaro Christian Medical University College, Moshi, Tanzania

**Keywords:** DrInC, Swahili, validation, adaptation, psychometric properties

## Abstract

**Aims:** To develop Swahili versions of the Drinker Inventory of Consequences (DrInC) and evaluate its psychometric properties among a mixed population in Tanzania.

**Methods:** A Swahili version of the DrInC was developed by a panel of bilingual Swahili and English speakers through translation and back-translation. The translated DrInC was administered to a sample of Tanzanian injury patients and a sample of the general population. The validity and reliability of the scale were tested using standard statistical methods.

**Results:** The translated version of the DrInC questionnaire was found to have outstanding domain coherence and language clarity. The tested scale and subscales have adequate reliability (>0.85). Confirmatory factor analysis (CFA) confirmed the five-factor solution by yielding adequate results. DrInC score is statistically significantly correlated with alcohol consumption quantity and the AUDIT score, suggesting that DrInC is able to predict alcohol use as well.

**Conclusions:** This study presents the first validation of the DrInC questionnaire with injury patients and a general population and the first adaptations of the DrInC questionnaire in the Tanzanian and Swahili setting. DrInC instrument was found to have satisfactory psychometric properties, resulting in a new medical and social research tool in this setting.

## Introduction

Alcohol is one of the leading causes of death, disease, and disability globally; alcohol is associated with more than 200 types of diseases in the International Classification of Diseases-10 (ICD-10), including injury, gastrointestinal diseases, cancer, cardiovascular diseases, alcohol use disorder (AUD), and fetal alcohol syndrome (1). Moreover, alcohol consumption accounts for 5.1% of the global burden of disease and injury disability-adjusted life years (DALYs) (2). Specifically, within Africa, alcohol accounted for 6.4% of all deaths and 4.7% of all DALYs in 2012 (3). The Kilimanjaro region, which includes Moshi, has one of the highest reported alcohol intake per capita in Tanzania (4, 5). The prevalence of AUD (define by CAGE score 2–4) in Moshi has been found to be 22.8% in men, 7.0% among women with partners, 9.5% among single women, 37.3% among female bar/hotel workers (4, 6).

Given the importance of injury in Tanzania, developing an alcohol consequence assessment tool is vital in both clinical practice and public health research. The Drinker Inventory of Consequences (DrInC) has been under development since 1989 (7). DrInC is a 50-item harm assessment questionnaire, which is used specifically for assessing adverse consequences of alcohol abuse. The DrInC has been validated in the US (8, 9). However, DrInC has not been cross-culturally validated nor psychometrically evaluated in Tanzanian culture or injury population.

Kilimanjaro Christian Medical Center (KCMC) Emergency Department (ED) data show ~30% of the injury patients consumed alcohol at the time of injury (10). Compared with patients present at a primary health care facility, those presenting in ED are more common to report higher problem drinking rate and alcohol dependence (11). Therefore, selecting injury patients as part of the study population may increase the sensitivity of this study because they are more likely to suffer from alcohol-related consequences than other populations.

Given the severity of injury in sub-Saharan Africa and the significant contribution of alcohol use to injury, it is important to understand the association between alcohol use and injury (12). To our knowledge, tools used to measure alcohol consequence is unavailable in most African countries. Thus, it is urgent to develop such objective measures in sub-Saharan Africa, especially among injury patients. One previous study has validated the Alcohol Use Disorders Identification Test (AUDIT) and CAGE questionnaire in Swahili, the primary language of Tanzania (13). This study aims to develop the first translation and adaptation of DrInC in Swahili and analyze its psychometric properties in Tanzania injury patients, including reliability and external validity.

## Materials and methods

### Study setting

Moshi is located in the Kilimanjaro Region of Northern Tanzania with over 180,000 people (14). The majority of people in Moshi are members of the Chagga, Pare, and Maasai ethnic group (14). Kilimanjaro Christian Medical Center (KCMC), the third largest hospital in Tanzania, is located in Moshi. KCMC is also a referral hospital for over 15 million urban and rural people in Northern Tanzania.

### Participants

Participants were composed of two independent samples: (1) a total of 341 injury patients and (b) a random sample of 500 adults in Moshi, Tanzania. This study included those who consumed alcohol at least once in their lifetime, 246 (72%) injury patients and 379 (76%) from the general population. Injury participants were included if they were ≥18 years old, seeking care at KCMC Emergency Department for an injury of any severity, clinically sober at the time of enrollment, medically stable, able to communicate in fluent Swahili, and consented to participate before discharge from the hospital. The general validation population was recruited from people on the hospital grounds (not patients but family members) and different random public locations in Moshi Urban, and all participants provided informed consent.

### Instruments

The DrInC is a 50-item harm assessment questionnaire, which is used specifically for assessing adverse consequences of alcohol abuse. Forty-five items are scored in a positive direction to measure the severity of alcohol problems, and 5 reverse-scaled control items are included (7). DrInC measures five categories: Interpersonal, Physical, Social, Impulsive, and Intrapersonal aspects, as indicated in Table 1 (7). Each category employs a time-frame focusing on the past 3 months, as well as, a lifetime measure of alcohol consequences. The negative consequences identified using DrInC have been shown to correlate with other outcome measures, such as psychosocial functioning and psychiatric dysfunctions (15).

**Table 1:** Summarizes the consequences by each subscale in DrInC.

**Subscale**	**Number of items**	**Description**	**Sample item**
Physical	8	Reflect acute and chronic adverse physical states resulting from excessive drinking	My physical appearance has been harmed by my drinking
Intrapersonal	8	Subjective perceptions	I have felt guilty or ashamed because of my drinking
Social responsibility	7	Consequences observable by others	I have had money problems because of my drinking
Interpersonal	10	The impact of drinking on the respondent's relationships	While drinking, I have said harsh or cruel things to someone
Impulse control	12	Impulsive actions, risk-taking, exacerbation of other substance use and legal problems	I have been overweight because of my drinking

The validation of DrInC also involved the AUDIT. AUDIT is an instrument used to identify people with problem drinking patterns (16). The 10-item AUDIT assesses alcohol intake, alcohol dependence, and alcohol-related problems. AUDIT's score ranges from 0 to 40, a score of 8 or more indicates harm drinking (17, 18). AUDIT's psychometric properties have been validated in many different regions, including Tanzania (13, 19–25).

## Ethical statement

This study was approved by the Institutional Review Board of the Duke University (IRB #Pro000061652) and Kilimanjaro Christian Medical Center Ethics Committee, as well as the National Institute of Medical Research in Dar Es Salaam, Tanzania.

### Translation and adaptation

Translation, adaptation, and content validation process have been overseen by a translation and cross-cultural adaptation committee (5 physicians, nurses, and researchers). Pilot surveys were administered among a convenience sample of 20 Tanzanian adults to evaluate the quality of questions and coherence of language, as well, as clarity and comprehension.

Independent back translation methods recommended by WHO were used during the translation of the instrument (26). Firstly, the DrInC questionnaire was translated by a Swahili translator. Followed by the back-translation process done by another bilingual translator. Then both translated versions were checked by four independent bilingual research nurses for discrepancies. Semantics issues were adjusted by the researchers' and the judges' committee.

For evaluating the consistency, a five-point Likert scale was employed to verify: (a) practical relevance, (b) language clarity of the translated instrument, and (c) theoretical coherence of the item. The judges' opinions were collected individually and later discussed jointly in a focus group to find discordances and improve the quality of the translation.

### Data collection

Patients presenting to the KCMC Emergency Department for an acute injury were screened for participation in our project. After an informed consent, they were surveyed prior to discharge from the hospital. DrInC and AUDIT questions were administered at the bedside as a part of the 45 min baseline survey. Data were collected by hand and entered into an Internet-based dataset (REDcap) with a quality control process conducted by the principal investigator (CAS) (Data Sheet in Supplementary Material). The general sample was collected by recruiting and consented random people (not patients but family members) around the hospital and different random public locations downtown.

### Data analysis

Sociodemographic data were presented as means with standard deviations and frequencies. All data analyses were conducted with R software. The missing data for the AUDIT and DrInC scales were imputed by using the multiple imputation method provided by the mice package (27). A sensitivity analysis showed there are no significant differences to the models with and without the imputed data.

### Reliability

Reliability is the overall consistency of a measure to produce consistent results in different settings. Different coefficient has its strengths and limitations. For example, unlike coefficient alpha, with congeneric items with uncorrelated errors, coefficient omega remains unbiased (28). Therefore, we measured the different indicators of reliability to check the DrInC items' homogeneity. The Cronbach's alpha was used to measure the internal consistency. Composite reliability (CR) and McDonald's Omega coefficient were also calculated based on CFA results.

### Evidence of validity

Confirmatory factor analysis (CFA) was conducted to test the internal structure of the DrInC based on the literature separating it into five domains (7). CFA model adequacy was tested by Weighted Least Square Means and Variance Adjusted (WLSMV). Average variance extracted (AVE) was tested, value above 0.5 was considered acceptable for convergent validity (29). The model adjustment was tested by fit indices: Root Mean Square Error of Approximation (RMSEA < 0.05, I.C. 90%), Chi-square (*X*^2^ and *P*-value), comparative fit index (CFI > 0.95), and Tucker-Lewis index (TLI > 0.95), as suggested in the literature (26). The above indexes were used to assess the degree of models fit the data (30).

The AUDIT is an instrument used to identify people with problem drinking patterns (16). Since the AUDIT has been validated with the patient population (13) and was frequently used together with the DrInC to identify alcohol problems (31, 32), moderate correlation between the DrInC and the AUDIT can also use as an evidence of concurrent validity. Spearman correlation between the audit score and the DrInC are reported along with a comparison of the DrInC scores according to the volume of drinking per drinking event. The ROC curve was also drawn to evaluate the predictive validity of the DrInC for using it to predict AUDIT categories [the cutoff point at 8, see Conigrave, Hall (18)].

## Results

### Sample characteristics

Our entire sample size is 626 (246 injury patients and 380 general population) and this study only included those who consumed alcohol at least once in their lifetime. Table 2 shows the sociodemographic profiles if the validation sample. Most of the participants were male (60%). The average age was 41.73 years old (*SD* = 23.86). Seventy-four percent (*n* = 463) of them reported consuming alcohol in the 12 months prior to the study. Among them, 52.3% consumed alcohol at least two times a week and 10.4% reported consumed at least 5 drinks per drinking day.

**Table 2:** Sociodemographic profile of the validation sample, *n* = 626.

**Variables**	
Age (years), mean (SD)	41.73 (23.86)
Male, N (%)	375 (60%)
Consumed alcohol in the last year, N (%)	463 (73.96%)
Consumed alcohol at least two times a week, N (%)	242 (52.3%)
Consumed at least 5 drinks per drinking day, N (%)	48 (10.4%)

### Descriptive characteristics

Table 3 provides means, standard deviations, and decile scores for the full-scale DrInC (45 items) the five DrInC subscales. It should be noted that all scores are skewed to the right, which suggests alcohol problems are concentrated in a small group of participants and the majority of our sample are generally free of alcohol problems even though they have consumed alcohol. The AUDIT median sum score was found to be lower than the cut-off point (< 8), suggesting the majority of our sample did not use alcohol harmfully. The DrInC median sum score of 2 suggests approximately half of the sample have less than two alcohol-related consequences.

**Table 3:** Sociodemographic profile of the validation sample.

	**AUDIT full scale**	**DrInC full scale**	**Physical subscale**	**Intrapersonal subscale**	**Social subscale**	**Interpersonal subscale**	**Impulse subscale**
Mean	9.06	7.45	1.43	2.15	1.3	1.33	1.22
SD	6.91	10.05	1.99	2.43	1.93	2.43	2.06
**DECILE**							
5th	2	0	0	0	0	0	0
10th	2	0	0	0	0	0	0
25th	3	1	0	0	0	0	0
50th	7	2	0	1	0	0	0
75th	12	10	3	3	2	2	2
90th	19.5	25	5	7	5	5.2	4
95th	22	30	6	7	6	7	6

### Translation and adaptation

All items were found to have language clarity and reliability coefficients above 0.8 for the DrInC full scale and five subscales (Table 4). These results suggest that the translated version of the DrInC questionnaire can be understood in Tanzanian culture.

**Table 4:** Reliability indicators.

	**DrInC full scale**	**Physical**	**Intrapersonal**	**Social**	**Interpersonal**	**Impulse**
Cronbach's alpha (CI 95%)	0.96 (0.96; 0.97)	0.83 (0.80; 0.85)	0.86 (0.85; 0.87)	0.85 (0.85; 0.85)	0.90 (0.89; 0.91)	0.82 (0.82; 0.84)
Omega 6	0.98	0.89	0.93	0.89	0.93	0.89
Composite reliability	0.99	0.94	0.96	0.95	0.97	0.95
Average extracted variance	0.71	0.69	0.74	0.75	0.78	0.62

### Reliability and internal structure

**Figure 1:**
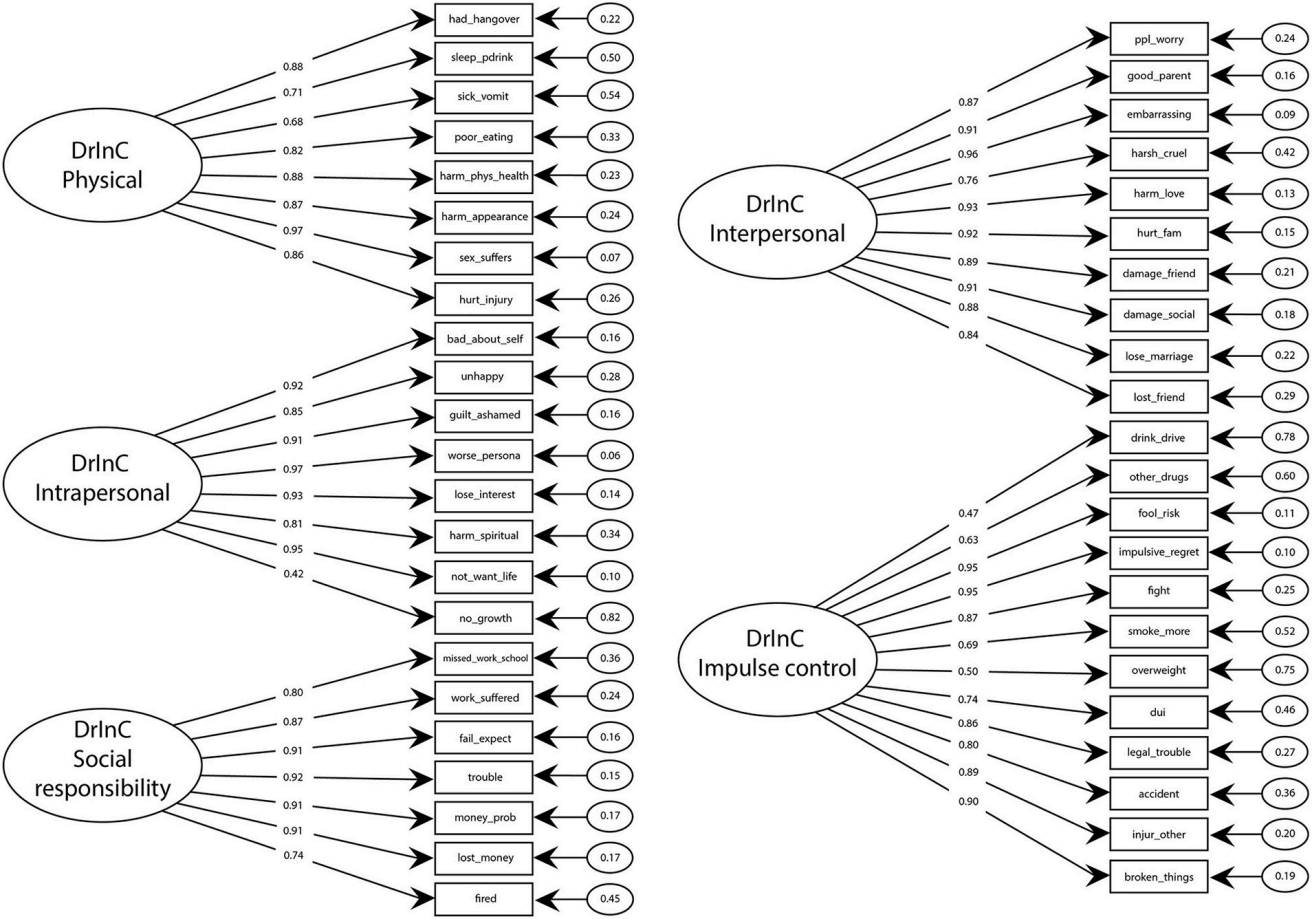
Confirmatory factor analysis diagram, factor loadings for the DrInC's five-factor model.

All reliability values range above 0.80 indicates that the DrInC scale and subscales have adequate reliability and internal consistency. DrInC's five-factor original CFA model showed satisfactory fit indicators and individual item reliability (Table 5, Figure 1). Figure 1 showed all items have factor loadings ranging from 0.42 to 0.97. The average extracted variances were all above 0.6 and were higher the cutoff at 0.5 in the literature (33). However, the modification index of “My sex life has suffered because of my drinking” variable was found be unusual high (100.9) and suggested it would perform better in the interpersonal subscale than in its original physical subscale. Therefore, we adapted the variable in our new model and found the adapted model fits better than the original one.

**Table 5:** Confirmatory factor analysis model fit indicators.

**CFA:**	**DrInC original scale**	**DrInC adapted**	**DrInC adjusted**
X^2^ (Df)/*P*-value	1781.34 (935)/0.001	1670.70 (935)/0.001	1472.93 (933)/0.001
RMSEA (CI 95%)	0.038 (0.035; 0.041)	0.035 (0.033; 0.038)	0.030 (0.027;0.033)
TLI	0.996	0.997	0.998
CFI	0.996	0.997	0.998

Spearman correlation coefficients were computed for the DrInC subscales and are displayed in Table 6. Correlation coefficients for the DrInC subscales are consistent and coefficients ranged between 0.7 and 0.8. Because correlations between subscales and certain items are high, we adjusted our model by linking variables that produce high residuals due to their high correlations. “My sex life has suffered because of my drinking” and “My marriage or love relationship has been harmed by my drinking” were linked and “I have had an accident while drinking or intoxicated” and “While drinking or intoxicated, I have been physically hurt, injured, or burned” were linked in the adjusted model. The adjusted CFA model performs better in terms of RMSEA, TLI, and CFI than the original and adapted models.

**Table 6:** ROC curve values and intercorrelations of DrInC subscales.

	**DrInC**	**Physical**	**Intrapersonal**	**Social**	**Interpersonal**	**Impulse control**
Sensitivity	52.00%	61.00%	55%	44.70%	47.50%	57.00%
Specificity	77.00%	62.60%	71.80%	82.80%	77.30%	71.20%
AUC	0.677	0.648	0.655	0.651	0.64	0.656
**CORRELATION ALPHA**						
Physical	–	–			
Intrapersonal	–	0.77	–		
Social	–	0.75	0.79	–	
Interpersonal	–	0.71	0.73	0.77	–
Impulse control	–	0.76	0.73	0.77	0.72	–

### Validity evidence

**Figure 2:**
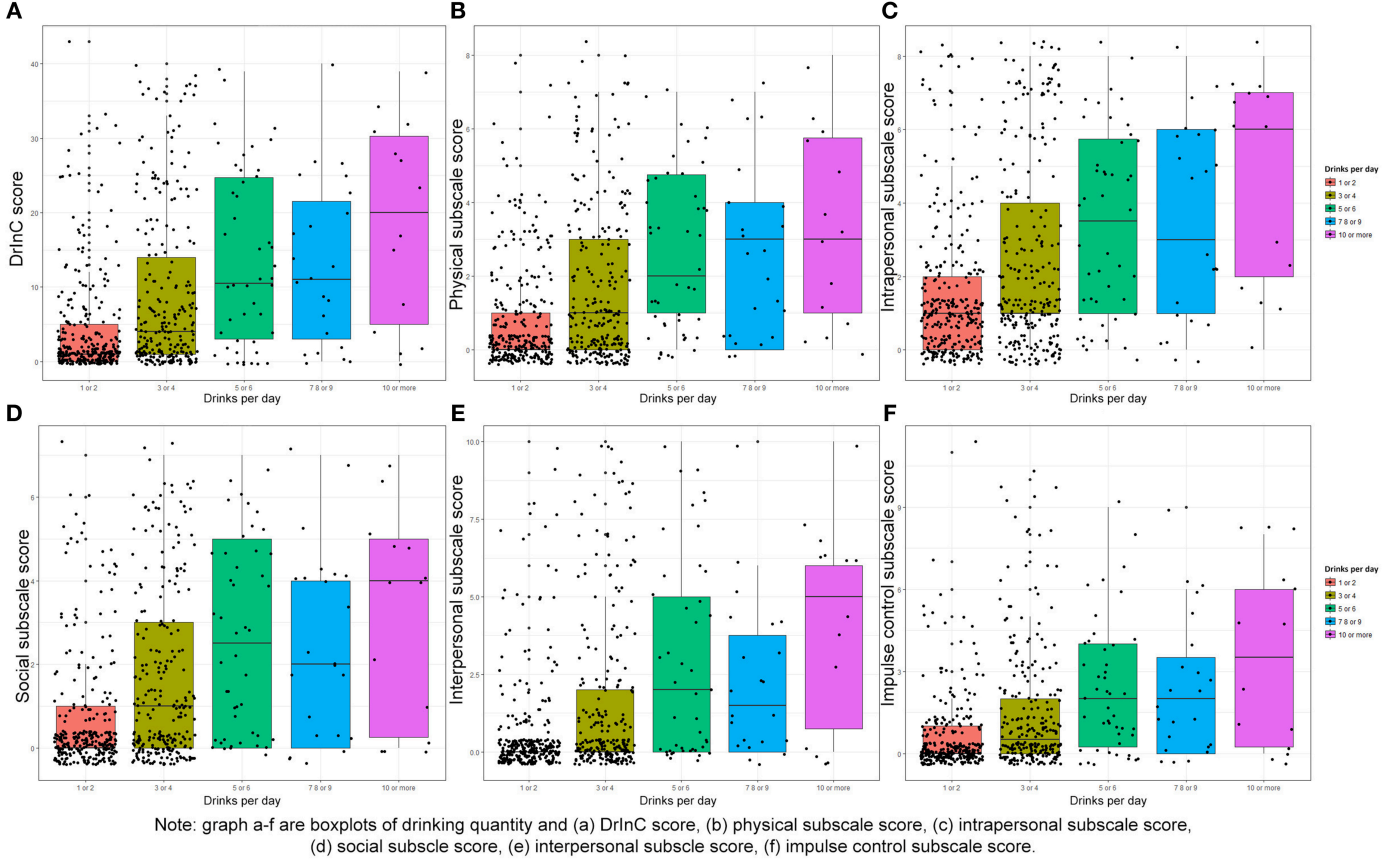
Box plot of drinking quantity and DrInC and its subscale scores. Graph A–F are boxplots of drinking quantity and **(A)** DrlnC Score **(B)** physical subscale score **(C)** intrapersonal subscale score, **(D)** social subscale score **(E)** Interpersonal subscale score, **(F)** impulse control subscale score.

**Figure 3:**
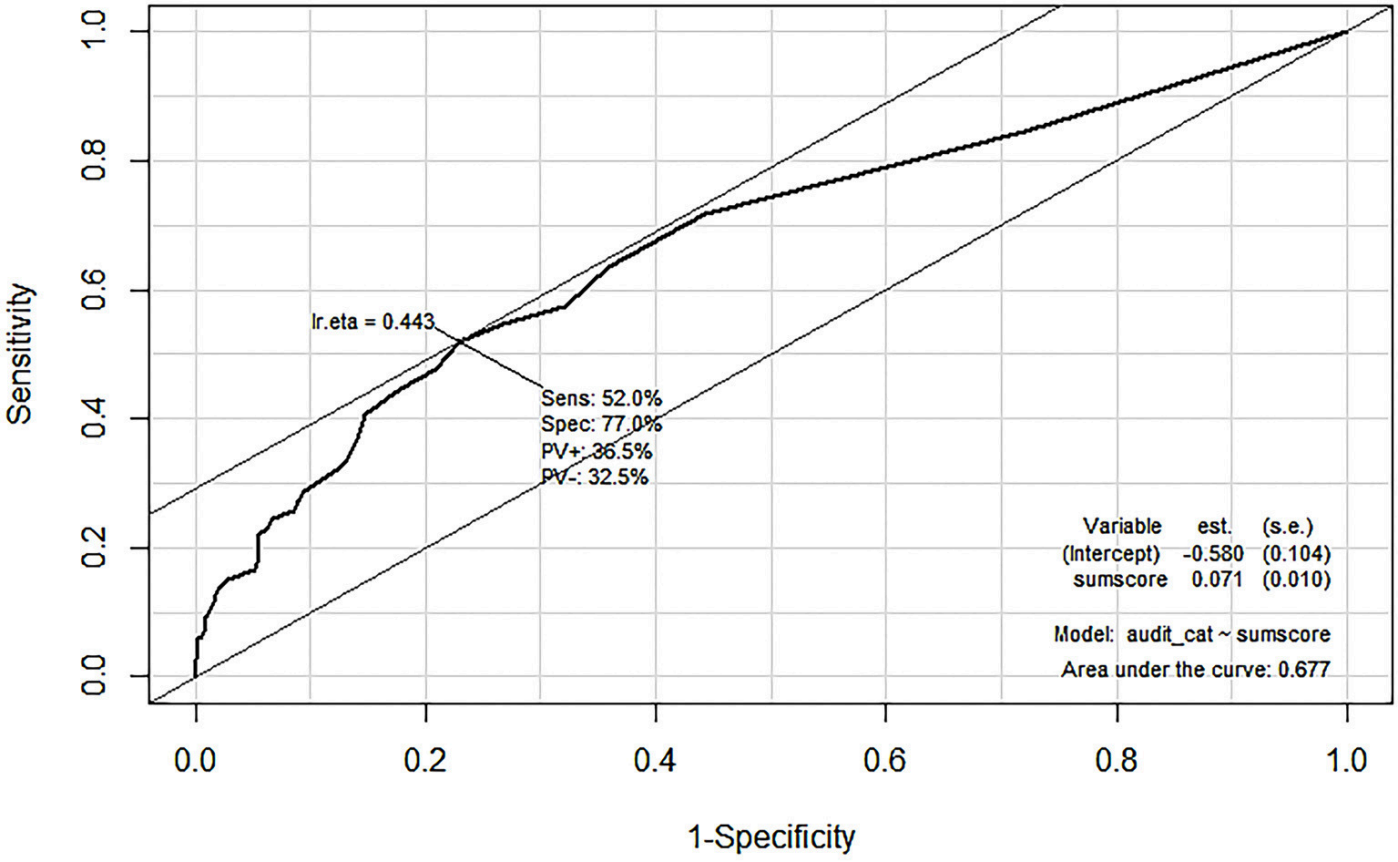
Using ROC curve to find the best DrInC cutoff point to predict AUDIT.

Figure 2 suggests strong positive correlations between the amount of alcohol people drink on a typical day and DrInC score (Pearson's coefficient of 0.64, *p* < 0.001) and its subscale scores. The DrInC and the AUDIT scores also have a strong correlation, with the polychoric correlation coefficient of 0.47 (*p* < 0.001). Figure 3 shows the DrInC cutoff point at the score of 6 yielded the best sensitivity and specificity of 0.52 and 0.77, respectively, when used predicting the AUDIT category if we select AUDIT cutoff point at 8. In other words, a person needs to have 6 consequences to be classified as a harmful alcohol user (with an AUDIT score no < 8). ROC curve values for subscales are displayed in Table 6. In general, specificity values were higher than sensitivity values in ROC curves.

## Discussion

This is the first project to provide validation for the DrInC, a five-dimensional tool designed to capture a broad range of alcohol-related consequences in Swahili and the Tanzanian culture. The DrInC questionnaire was originally designed with the physical, intrapersonal, social, interpersonal, and impulse control dimensions which have been supported in other studies (7, 8).

To our knowledge, DrInC has not been previously validated in Tanzanian culture. This is the first study to adapt DrInC cross-culturally to Swahili in Tanzania, or to a mixed population in sub-Saharan Africa. This study also examined several measures of validity and reliability to explore the psychometric properties of DrInC. The confirmatory factor analysis' results suggest the translated version of DrInC performs well in five-dimensional models and display similar psychometric properties regarding other population and languages (8, 9). Therefore, the fact that no further changes were needed suggests that the Swahili version of DrInC can be used to assess negative alcohol consequences in this particular population.

We found DrInC and AUDIT scores distribution were obviously skewed to the left. These skewed distributions might attribute to our mixed selection of a small portion of injury patients who are more likely to suffer from alcohol problems and the large portion of the general population who are more likely to be free from alcohol problems.

It should be noted that the correlation between subscales within in DrInC is high, as reported previously in the literature (8, 9). This might indicate that these subscales are closely linked. It could be explained by the possibility that these alcohol consequences have multiple shared determinants rather than individual determinants. For instance, it is common for someone who has had a fight and at the same time he got injured. Thus, he would be both positive on the DrInC's impulse control consequences and physical consequences. We adjusted the original CFA model by incorporating items that were closely related in the scale to the model and yielded better fit indexes.

The DrInC full scale and subscales were found to have acceptable internal reliability and consistency. Multiple coefficients were used rather than the Cronbach's alpha itself because Cronbach's alpha has been criticized since it would produce lower value than composite reliability (CR) when items are congeneric (28). Thus, less biased CR and Omega coefficients were also calculated. All values are high and consistent throughout the analysis, suggesting the Swahili translated version has good reliability. In spite of this, the original CFA model was not perfect and we adapted and adjusted the CFA model to make the model performs better. Our adapted model suggested the “My sex life has suffered because of my drinking” variable might be categorized in the interpersonal subscale instead of its original physical subscale in the Swahili version of DrInC.

This is also the first study to test the correlation between the DrInC and the AUDIT and to use the DrInC to predict the AUDIT. Since both DrInC and AUDIT are important alcohol use and consequence measurement tools. A moderate or strong association provides evidence of the external validity of alcohol outcome measurements. We found the cutoff point of 6 consequences produce the best sensitivity and specificity of 0.52 and 0.77, respectively, when used to predict AUDIT score of 8 or more. All specificity values in all ROC curves were found to be higher than sensitivity values, suggesting DrInC can be very specific when detecting people with drinking problems while may not be as good in distinguishing drinkers. In addition, the DrInC score was noted to be correlated with alcohol consumption quantity, suggesting that DrInC is able to discriminate the alcohol use quantity as well.

Two limitations of this study should be considered. One is our specific sample. The participants of this study were drawn from injury patients presenting to a hospital for care and a population-based cohort. The injury population is made up of patients that are able to survive to reach care in a limited resource setting, survive with a relatively good physical function to be able to answer a verbal questionnaire and provide informed consent, but this is representative of the overall injury population in this setting (34). We deliberately oversampled injury patients because they have more alcohol-related consequences than the general population as mentioned previously. This study is an important step to understand the alcohol use consequences and injury in Tanzania. However, our mixed participants are unlikely to represent the whole of the Tanzanian population. Therefore, further validation of DrInC is needed when using this tool in other areas of Tanzania.

The second limitation is the absence of criterion validity in the analysis, which allows calculating sensitivity, specificity and cutoff point. This study is aimed to provide evidence for psychometric properties of the translated version of DrInC, therefore we did not test the criterion validity. We hope the criterion validity can be addressed in the future study.

In conclusion, this study shows the first validation of the Swahili version of DrInC for injury patients in Tanzania. The DrInC is a reliable, valid, and clinically useful tool for clinicians and researchers to measure the adverse consequences of drinking in this setting. Although this study examined psychometric properties of DrInC in Swahili, DrInC has not been validated in other sub-Saharan African languages and communities. Therefore, we hope to see more research on validating this useful tool in other settings across sub-Saharan Africa.

## Author contributions

DZ, CS, BM, and JV developed the conceptual question and rationale for this project. BM and CS were responsible for data collection. DZ, JV, and QH were responsible for the data analysis and graphing. DZ, JV, and CS contributed to the interpretation of the results. DZ wrote the initial draft of the manuscript. All authors critically edited and approved the final manuscript.

### Conflict of interest statement

The authors declare that the research was conducted in the absence of any commercial or financial relationships that could be construed as a potential conflict of interest.
